# Algorithmic Generation of Parameterized Geometric Models of the Aortic Valve and Left Ventricle

**DOI:** 10.3390/s25010011

**Published:** 2024-12-24

**Authors:** Nikita Pil, Alex G. Kuchumov

**Affiliations:** 1 Biofluids Laboratory, Perm National Research Polytechnic University, 614990 Perm, Russia; targs2@gmail.com; 2 Department of Computational Mathematics, Mechanics and Biomechanics, Perm National Research Polytechnic University, 614990 Perm, Russia

**Keywords:** aortic valve, left ventricle, parameterized geometry, synthetic data

## Abstract

Simulating the cardiac valves is one of the most complex tasks in cardiovascular modeling. As fluid–structure interaction simulations are highly computationally demanding, machine-learning techniques can be considered a good alternative. Nevertheless, it is necessary to design many aortic valve geometries to generate a training set. A method for the design of a synthetic database of geometric models is presented in this study. We suggest using synthetic geometries that enable the development of several aortic valve and left ventricular models in a range of sizes and shapes. In particular, we developed 22 variations of left ventricular geometries, including one original model, seven models with varying wall thicknesses, seven models with varying heights, and seven models with varying shapes. To guarantee anatomical accuracy and physiologically acceptable fluid volumes, these models were verified using actual patient data. Numerical simulations of left ventricle contraction and aortic valve leaflet opening/closing were performed to evaluate the electro-physiological potential distribution in the left ventricle and wall shear stress distribution in aortic valve leaflets. The proposed synthetic database aims to increase the predictive power of machine-learning models in cardiovascular research and, eventually, improve patient outcomes after aortic valve surgery.

## 1. Introduction

The prevalence of aortic valve replacement surgeries due to stenosis has been steadily increasing each year, reflecting the growing burden of cardiovascular diseases in the aging population [1,2,3]. More than 10% of individuals over the age of 70 are affected by aortic stenosis, a condition characterized by the narrowing of the aortic valve, which can lead to severe health complications and requires timely surgical intervention [4]. The disease remains the most common valvular heart pathology in developed countries, with prevalence rising markedly with age, affecting approximately 2–4% of those over 75 years old and reaching about 3.4% in Europe and North America. In the U.S., about 1.5 million patients have been diagnosed, and nearly 500,000 presents with severe symptomatic disease requiring treatment [5]. Until recently, surgical aortic valve replacement (SAVR) was the primary therapy, accounting for roughly 50,000–60,000 procedures annually [6,7]. The introduction of transcatheter aortic valve replacement (TAVR) since the mid-2000s has led to a marked increase in interventions, growing from about 5000 procedures in 2012 to over 70,000 by the late 2010s, with global numbers exceeding 200,000 [8]. In Europe, countries such as Germany reported a surge in TAVR procedures from a few thousand to more than 18,000 per year. France, Italy, and the U.K. also report annual volumes rising from hundreds to several thousand [9].

Japan, which granted regulatory approval for TAVR in 2013, now reports several thousand procedures annually, demonstrating the global trend toward a more rapid and widespread adoption of transcatheter interventions [10,11,12].

The accurate prediction of the long-term outcomes of these surgical interventions is critical for improving patient care and optimizing treatment strategies. This prediction relies heavily on the biomechanical modeling of hemodynamics within the aortic valve, a complex and resource-intensive task [9]. Biomechanical models are sophisticated and demand substantial computational capacity for real-time application, which is crucial for clinical decision-making [10,11,12]. To address this, machine-learning (ML) methods have emerged as a promising solution, offering the potential for rapid and accurate predictions [13,14]. The effectiveness of ML models in this context is intrinsically linked to the quality and comprehensiveness of their training datasets [15,16].

This study focuses on generating a synthetic database of geometric models of the left ventricle and aortic valve leaflets. Currently, there are algorithms capable of processing multislice computed tomography (MSCT) data and converting them into three-dimensional models through segmentation techniques that already exist [17,18,19].

However, creating a large-scale database through this method would require performing tomography scans on thousands of individuals, followed by extensive data processing, which is impractical. An alternative approach involves the creation of synthetic geometries, enabling the generation of a diverse array of geometric models of the human aortic valve and left ventricle with varying shapes and sizes.

Several methodologies have been proposed to present the geometry of the aortic valve leaflets. For example, Hsu et al. utilized T-splines to develop parameterized geometries, allowing for flexible and detailed representations of the valve structure [20]. Hoejimakers et al. described a statistical shape model of the aortic valve based on 74 iso-topological geometries, providing a comprehensive framework for understanding the valve morphology [21]. Similarly, Bosmans et al. applied statistical modeling to the geometry of the aortic root using data from 89 patients [22]. Hagenah et al. introduced a method for assessing leaflet shapes based on the ultrasound diagnostic data, further contributing to the repertoire of techniques available for aortic valve modeling [23].

**Figure 1:**
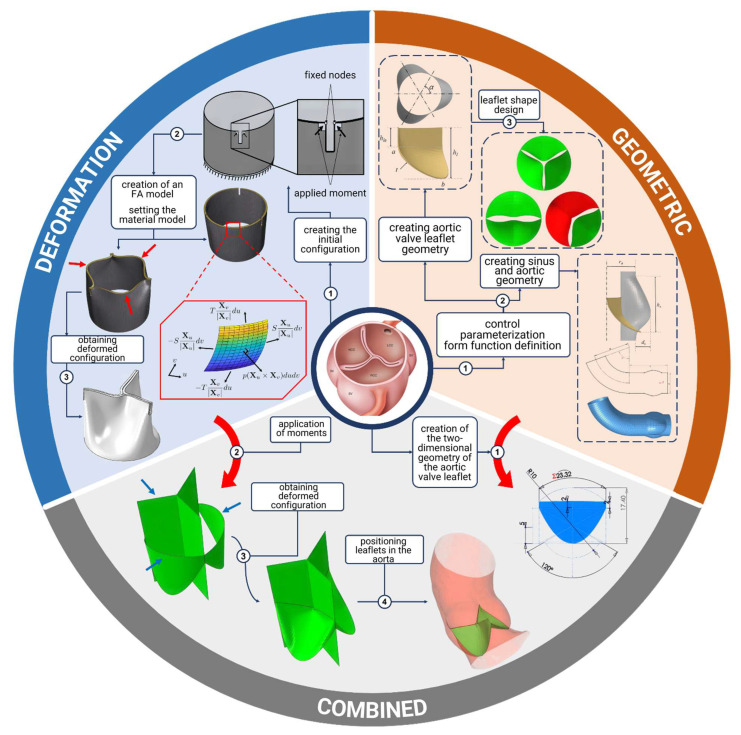
Aortic valve geometry model approaches.

In modeling hemodynamics and examining the mechanical behavior of the aortic valve, it is essential to create geometric models based on physiological data and the accumulated statistical information on the shapes and sizes of the aorta and its leaflets. As illustrated in Figure 1, two primary approaches can be employed to construct such models: geometry-parameterized models and deformation-based models. In geometry-parameterized models (Figure 1, top-right), several controlling parameters and functions are used to define the leaflet’s curvature [24,25,26]. A similar process is used to define the geometry of the aortic root. By adjusting these parameters, researchers can generate a wide range of geometric models with various shapes and sizes. A critical aspect of this approach is determining the appropriate range of parameter variations and developing a comprehensive set of functions. The second approach (Figure 1, top-left) focuses on constructing geometric models by deforming an initially idealized leaflet configuration from a fully open state to a closed position [27,28]. Typically, a cylindrical blank is created and divided into three sections that are fixed at certain nodes. A load is then applied to each of these sections until they come into contact, and the resulting configuration serves as the baseline model of the aortic valve leaflets. Although this method involves fewer parameters, it is strongly dependent on the choice of the material model used to describe the mechanical properties of the valve leaflets. Combined approaches (Figure 1, bottom) that involve specifying the geometric dimensions of a two-dimensional leaflet and determining its curvature through deformation to make contact with the remaining leaflets are also employed [29]. 

This paper proposes an approach to creating a synthetic database of geometric models. While existing MSCT data-processing algorithms allow three-dimensional models to be generated via segmentation, it is impractical to scale them to a large database. Therefore, we propose the use of synthetic geometries that allow the creation of a variety of aortic valve and left ventricular models with different shapes and sizes. This comprehensive synthetic database is intended to enhance the predictive capabilities of ML models in cardiovascular research and, ultimately, improve the outcomes of aortic valve surgery patients.

## 2. Materials and Methods

### 2.1. Aortic Valve Geometry Generation

The aortic valve consists of three leaflets: the right coronary, left coronary, and non-coronary leaflets. The sizes and shapes of these leaflets vary among individuals, necessitating the identification of control parameters that can accommodate the full spectrum of aortic valve types. According to the literature, the circumference of the aortic annulus in adults ranges from 4.7 to 9.4 cm (commonly 6.9–7.2 cm) and from 1.9 to 6.3 cm (commonly 2.2–4 cm) in children. The diameter of the aorta varies from 1.5 to 3 cm (commonly 2–2.3 cm) in adults and from 0.6 to 2 cm (commonly 0.7–1.3 cm) in children.

The height of the aortic bulb reaches 17–25 mm in adults and 6–14 mm in children. Within the aortic bulb, there are three depressions known as the sinuses of Valsalva, with heights ranging from 1.7 to 2 cm in adults and 0.6 to 1.4 cm in children. The depth of these sinuses varies from 1.5 to 3 mm. To create detailed leaflets of the aortic valve, we begin by defining key parameters such as the radius of the aorta and the height of the leaflet. The shape of each leaflet is controlled using guiding curves, which are mathematically described by logarithmic functions. This approach ensures that the variability in leaflet shapes is captured accurately.

**Figure 2:**
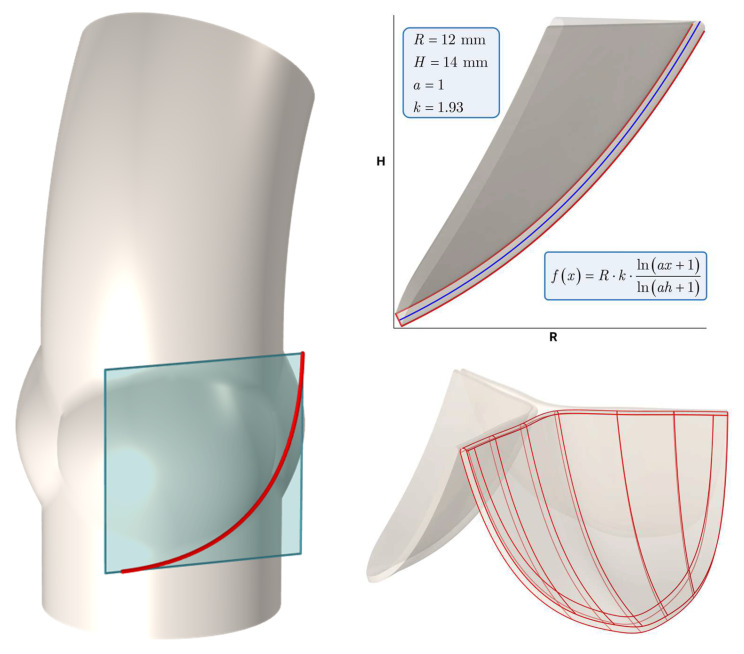
Leaflet shape configuration.

The algorithm for constructing an idealized geometric model involves several critical steps. First, the aortic root is segmented into three equal sectors, each representing one of the valve leaflets. A plane perpendicular to the radius of one of the sectors is constructed to define the generating curve. A generating curve is defined in this plane (Figure 2), for which the values of the following parameters are set: R is the aortic radius, H is the height of the leaflet, a modifies the curvature of the leaflet, and k is responsible for the shape of the free edge.

This plane is systematically rotated to define a series of generating curves that form the basis of the leaflet shape. By adjusting the parameters in the function equations, different aortic valve leaflet geometries can be obtained, allowing a wide range of models to be generated.

### 2.2. Left Ventricular Geometry Generation

In this study, we created 22 variations of the left ventricular geometry: one original model, seven models with varying heights, seven with varying wall thicknesses, and seven with differing shapes. Each model was validated using real patient data to ensure anatomical accuracy and physiologically normal fluid volumes. The primary objectives in constructing the geometry were to achieve an anatomically similar shape of the ventricle, ensure the fluid volume matched the literature data, and align the output orifice with the inlet orifice of the aortic geometry. The construction of geometric models of the left ventricle is primarily based on a geometric approach. The algorithm used to design models from two-dimensional echocardiographic data is adopted from [30]. The idea is to construct a central line and form key ellipses where significant changes in distances to the ventricle walls occur. By using the profile of the central line and a set of ellipses, the geometric model of the left ventricle is obtained through the lofting process. The geometry creation process involved several stages. Initially, echocardiography images of the left ventricle in two planes—frontal and sagittal—were obtained. The left ventricle was traced along the contours of both images, and planes were projected, which would subsequently become circles used to construct the computational model. For simplicity, it was assumed in the first approximation that the semi-major axes of the ellipses were equal. The model height was set at 80 mm, from which the circle sizes for construction were equivalently calculated. Using the loft function, the internal model of the ventricle was created. Similarly, the external model of the ventricle was constructed, with the circles increased by 10 mm each. The internal part was then subtracted from the external part, thereby creating a cavity for fluid calculation. The height of the left ventricle was varied between 73 mm and 89 mm. As the height changed, the volume of fluid inside the ventricle also varied within the physiologically normal ranges of the human left ventricle. To create different ventricle shapes, additional echocardiograms of the left ventricle were used. The original geometry was taken as a basis, and the centers of the circles were shifted along the x-axis; in addition, the sizes of some circles were adjusted. The wall thickness of the ventricle was varied in a similar manner.

### 2.3. Electrophysiological Model of Left Ventricle Contraction

The designed geometries of the left ventricle can be used to numerically simulate the connections between electrophysiology and ventricle contraction. Such simulations are very important in several clinical applications [31,32].

The propagation of the electrophysiological potential F in the myocardium is described by the following equation [33]:

(1) $$\chi_{m}C_{m}\frac{\partial }{\partial t}\Phi +\nabla \cdot \left(-D\nabla \Phi \right)+\chi_{m}I_{ion}(\Phi ,F,r_{i})=0,$$

where $\chi_{m}$ is the surface-to-volume ratio, m^−1^; $C_{m}$ is the membrane capacitance, F·m^−1^; $\chi_{m}C_{m}$ acts as the damping coefficient; and *Φ* is a potential. The conductivity tensor includes isotropic and anisotropic parts that depend on the fiber direction [34]:

(2) $$D=(d_{iso}C_{m}\chi_{m})I+\left(d_{ani}C_{m}\chi_{m}\right)a_{0}⊗a_{0},$$

(3) $$\Phi =\varphi \beta_{\varphi }+\delta_{\varphi },\;t=\beta_{t}\tau ,$$

(4) $$\beta_{t}=t_{\beta }\cdot \left(1-\tau_{0}\frac{t_{a}-t_{0}}{t_{1}-t_{0}}\right),\;t_{a}=t_{\alpha }\cdot \left(1-\frac{Z}{Z_{LV}^{apex}}\right),$$

The time-scaling parameter *β*_t_ is considered to be dependent on the activation time *t_a_*. During the cardiac cycle, the activation time (i.e., the time between depolarization and repolarization) is not constant throughout the myocardium. The areas that depolarize last are repolarized first. The parameters are chosen according to the experimental values of the resting potential of the heart, which is −80 mV, with a maximum potential value of 20 mV.

A dimensional representation of the ionic current equation is used to match the experimental values of electric potential and activation time in the myocardium.

(5) $$I_{ion}=C_{m}\frac{\beta_{\varphi }}{\beta_{t}}\left({\tilde{I}}_{e}+{\tilde{I}}_{m}\right).$$

The ionic current *I_ion_* is the sum of the excitation-induced (purely electric) current *I_e_* and the stretch-induced current *I_m_* [34]. To represent the purely electric part of the ionic current *I_e_*, the Aliev–Panfilov equation is used as a function of the electric potential [35].

(6) $${\tilde{I}}_{e}\left(\varphi ,r\right)=c\varphi \left(\varphi -\alpha \right)\left(\varphi -1\right)+r\varphi ,$$

(7) $${\tilde{I}}_{m}(F)=\theta G_{s}\left(\lambda \left(F\right)-1\right)\left(\varphi -\varphi_{s}\right),$$

(8) $$\frac{\partial r}{\partial \tau }=\left(\gamma +\frac{\mu_{1}}{\mu_{2}+\varphi }r\right)\left(-r-c\varphi \left(\varphi -b-1\right)\right).$$

The equation for the current induced by stretching is as follows:

(9) $$\frac{\partial S_{a}}{\partial t}=\varepsilon \left(\Phi \right)\left[k\left(\Phi -\Phi_{r}\right)-S_{a}\right].$$

Active stresses are added by the second Piola–Kirchhoff tensor in different proportions along fibers and normal to surface [33]:

(10) $$S=S+S_{a}\left[\eta_{1}\left({\bar{a}}_{0}⊗{\bar{a}}_{0}\right)+\eta_{2}\left({\bar{S}}_{0}⊗{\bar{S}}_{0}\right)+\eta_{3}\left({\bar{n}}_{0}⊗{\bar{n}}_{0}\right)\right].$$

Moreover, *ε(Φ)* is the delay function [34]:

(11) $$\varepsilon \left(\Phi \right)=\varepsilon_{0}+\left(\varepsilon_{0}+\varepsilon_{1}\right)\exp\left(-\exp\left(-\zeta \left(\Phi -\Phi_{t}\right)\right)\right)$$

### 2.4. Simulation of Opening/Closing of Aortic Valve Leaflets

The 3D FSI problem considering the opening and closing of aortic valve leaflets was solved to evaluate wall shear stress in leaflets. The problem statement, mechanical properties, and boundary conditions used to simulate leaflets’ mechanical properties can be found in our previous paper [36], and was employed to simulate mechanical properties of leaflets. The methodology developed can be used in clinical practice to predict the immediate and medium-term results of Ozaki operations [37,38,39].

## 3. Results

We have developed a software module that enables the creation of both single geometries with specified parameters and pseudo-randomized models of the aortic valve and left ventricle based on MSCT data. The key outcome of the program is the obtained geometries of aortic valve leaflets, which vary in curvature, size, and free edge shape. Similarly, 22 models of the left ventricle were generated with variations in height, wall thickness, and shape. The main parameters of the designed models are presented in Table 1.

### 3.1. Left Ventricles with Varied Heights

**Figure 3:**
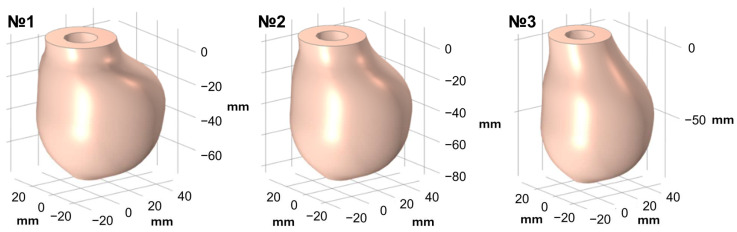
Three various geometries of left ventricle with various heights.

Left ventricular heights varied from 73 mm to 89 mm. As the height varied, the volume of fluid inside the ventricle also varied. The geometries are presented in Figure 3. The fluid volume varied within the physiologically normal volumes of the human left ventricle.

### 3.2. Left Ventricles with Varied Shapes

Other echocardiograms of the left ventricle were taken to create the ventricular shapes. This time, however, the original geometry was taken as a basis and the centers of displacement of the circles along the *x*-axis were changed, as well as the sizes of some circles. The volume varied within physiologically normal levels for the human left ventricle.

### 3.3. Left Ventricles with Varied Volumes

The wall thickness is the distance from the wall of the inner volumetric body (fluid cavity) to the wall of the outer volumetric body.

The design of a synthetic database of geometric models involves identifying control parameters that effectively capture the variability in the shapes and sizes of leaflets observed among different patients. This process entails generating a wide spectrum of synthetic geometric models by varying these control parameters. Deformation methods, incorporating material properties, are used to simulate the transition of leaflet shapes from fully open to closed states, enhancing the realism of the models. Validating these synthetic models against real patient data is crucial for ensuring their accuracy and reliability. Parameters and functions are iteratively refined to improve model precision.

### 3.4. Aortic Valve Leaflet Geometries

**Figure 4:**
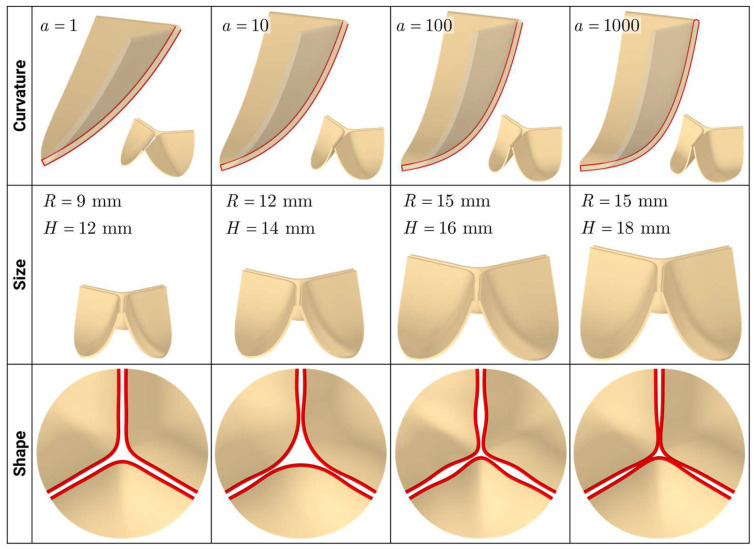
Aortic valve synthetic geometry models.

Figure 4 shows variations in aortic valve leaflet geometries depending on curvature, size, and shape. It can be seen that variations in different parameters can generate models of either older or younger patients’ valves. By applying this comprehensive approach, we aim to establish an extensive synthetic database of aortic valve geometries. This database will significantly enhance the capabilities of predictive modeling for surgical outcomes, utilizing advanced machine-learning techniques to provide more accurate and reliable predictions, ultimately improving patient care and treatment outcomes.

### 3.5. Comparison of Gemetric Models

To validate the geometric modeling algorithms for the aortic valve and the left ventricle, MSCT scans of six patients (four men and two women; see Table 2) were analyzed. The key dimensions included the radius and height of the aortic valve leaflets, as well as the height, radius, and wall thickness of the left ventricle. In addition, the volume of the ventricle was computed. All measured values fell within the designated modeling ranges. For the aortic valve, neither the leaflet radius nor height exceeded 14 mm. The left ventricle displayed a broader range of dimensions, with heights from 75 to 85 mm, wall thicknesses from 8 to 11 mm, and radii between 26 and 31 mm—values that were, in fact, smaller than those initially assumed in the model geometries.

**Figure 5:**
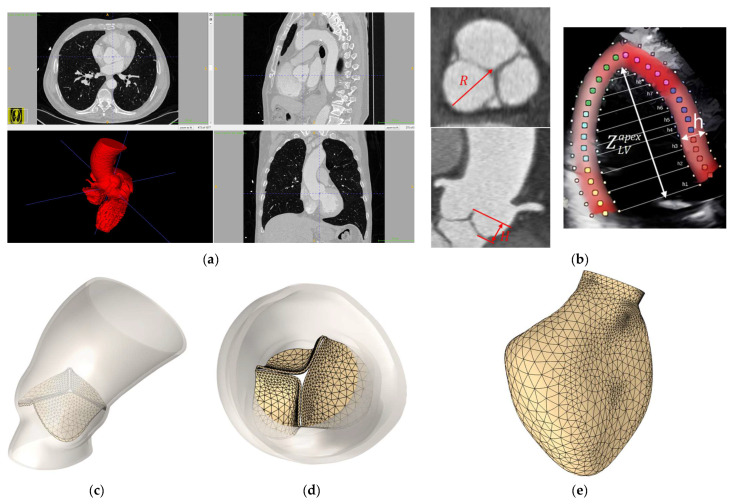
(**a**) Segmentation of aortic valve and left ventricle, (**b**) key measurements, (**c**,**d**) patient-specific aortic valve and root geometry model, and (**e**) patient-specific left ventricle geometry model.

We also constructed patient-specific models based on the segmentation of the MSCT scans of the aortic valve, aorta, and left ventricle (Figure 5) to visually compare the resulting geometries. Such an approach clearly enables the reproduction of detailed effects that can be crucial for understanding hemodynamic processes. Nonetheless, a simplified model geometry can still be employed to refine the overall research methodology. Moreover, they offer several advantages in terms of performing quantitative analyses and generating large databases of synthetic geometries.

### 3.6. Simulation of Left Ventricle Contraction

**Figure 6:**
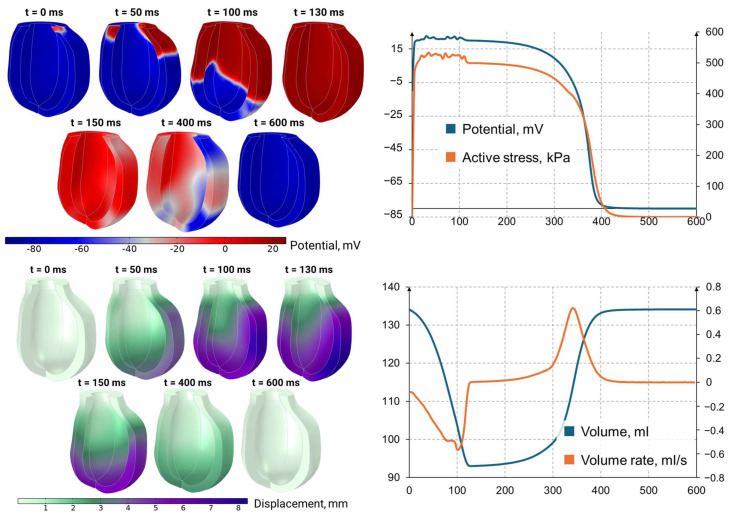
Electrophysiologic signal and displacement distribution during ventricle contraction.

**Figure 7:**
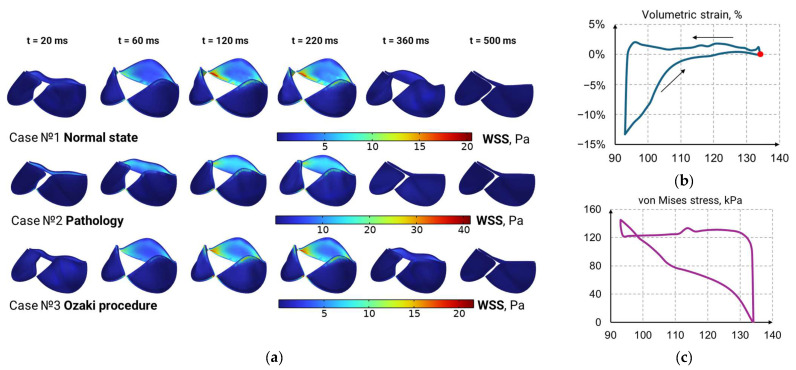
(**a**) Wall shear stress distribution in aortic valve leaflets, and (**b**,**c**) volumetric strain and von Mises stress loops for ventricle contraction.

Figure 6 and Figure 7 show the results of the left ventricle contraction simulations. Systole–diastole closed loops are used to plot the changes in ventricular volumetric strain and von Mises stress during the heart cycle. The dependence of the active potential corresponds to the data presented recently in [40]. It is believed that strain–volume dependence can be used as a noninvasive evaluation tool for dynamic heart function by combining systolic and diastolic biomechanical features. The simulated volumetric strain loops data are similar to the findings presented in [41]. In addition, it should be pointed out that the simulated von Mises stress results correspond to data presented by Dorri et al. [42], who noted that values vary around 100 kPa within a range of about 50 kPa.

### 3.7. Wall Shear Stress Distribution in Aortic Valve Leaflets

The wall shear stress distributions in aortic valve leaflets in a healthy state, in a pathological state, and after the Ozaki operation are shown in Figure 7. It can be seen that the stress distributions in case 1 and case 3 are similar. At the same time, the wall shear stress distribution in case 2 is two times higher than that in the healthy state. Elevated wall shear stress is observed in regions where leaflets attach to the fibrous annulus. Normal physiological levels of WSS within arteries range from 1 to 7 Pa in vivo, whereas the WSS in veins is lower and ranges from 0.1 to 0.6 Pa [43]. In the case of blood flow in the aortic valve, it was shown that the WSS changes from 0 to 20 Pa in the healthy state and from 0 to 40 Pa in the pathological state. In the case of blood flow in arteries, WSS may play a role in the pathogenesis of aneurysmal disease and atherosclerosis development. It is known that a lower WSS promotes atherosclerotic plaque development in blood vessels [44].

Table 3 compares the simulation results for the three cases under consideration with data reported in the literature, as well as with our previous two-dimensional study. In this comparison, the in vivo and in vitro experimental data are marked with “*”, and numerical experiment data are marked with “**”.

Under normal conditions, the modeled WSS values align well with the range reported in the literature (15–21.3 Pa). In the pathological state, the values increase, indicating an intensified mechanical influence, while, after the Ozaki procedure, they decrease slightly, in line with the expected improvement in flow parameters.

## 4. Discussion

In this study, software for the design and generation of synthetic geometries of the left ventricle and aortic valve leaflets was developed. As an example of our model’s application, a numerical evaluation of ventricle contraction was performed. An electrophysiological model was adopted to simulate the deformation behaviors of the ventricle. The model can be applied to the simulation of pathological states like heart failure or myocardial infarction in a large cohort of synthetic geometries.

### 4.1. Software for Cohort Geometries Design

Recently, machine learning and deep learning have become widely used in clinical practice [45]. The adoption of ML and DL tools in cardiovascular surgery is a cutting-edge technique that has a potential positive impact on surgery results [46]. The combination of numerical FE simulations with ML or DL analysis is a crucial task for obtaining adequate results [47]. As shown previously, ML can accelerate or substitute numerical simulations. Nevertheless, to produce adequate simulations, it is necessary to use large cohorts of geometries. Medical institutions usually have insufficient numbers of geometries, making the application of ML or DL infeasible. Therefore, the design of synthetic geometries can be an efficient way to supplement insufficient geometries. We hope that this study will enable the implementation of ML techniques to accelerate valve design in the simulation of left ventricles and aortic valve leaflets. It should be noted that the reconstruction of aortic valve leaflets from CT images is challenging because the thickness of the leaflets is small [48]. The method of designing aortic valve leaflet geometries presented in this paper has a lot of potential benefits. It can produce exact geometries similar to patient-specific ones, with similar dimensions. Moreover, using our method, the number of geometries needed for ML adoption can be generated automatically within a short period of time.

### 4.2. Left Ventricle Electrophysiology

When developing novel surgical and pharmaceutical approaches to treat and prevent infarction-induced heart failure, a noninvasive method for assessing ventricular contractility would be highly beneficial. Although our model can only realistically simulate a restricted range of regional left ventricular mechanics, it currently represents one of the most anatomically and functionally realistic models available. Further analysis of pressure–volume correlations and fiber stress distributions will provide valuable insights into the effectiveness of new cardiovascular techniques and technologies for the treatment of ischemic cardiomyopathies.

However, the use of generic fiber orientation maps remains a notable limitation of our method. The actual myofiber architecture varies significantly between individuals, influenced by factors such as age, pathologies, and specific anatomical characteristics [49,50]. By relying on a standardized representation rather than patient-specific data, the model may fail to capture subtle regional variations in fiber alignment, potentially leading to discrepancies between the predicted and observed outcomes and reducing the clinical relevance of the simulations. While recent advances have demonstrated the feasibility of in vivo diffusion tensor imaging, it remains challenging to densify such sparse datasets, and current approaches often rely on ex vivo measurements. Achieving a more personalized fiber orientation mapping would require advanced imaging techniques or computational methods capable of inferring detailed, patient-specific fiber distributions. Such progress would enhance the fidelity of model predictions and improve its utility in guiding clinical decision-making.

### 4.3. Wall Shear Stress Distribution in Aortic Valve Leaflets

Finding a relationship between WSS and aortic valve leaflet pathology is a major challenge. Using databases of geometries and performing a large number of simulations together with clinical observations can be considered as a first step in resolving this issue, but the problem persists that we still cannot interpret much of the results. The appropriate choice of criteria for the WSS value in the healthy and pathological states is still a puzzle requiring further research. The WSS distribution on the leaflets, which may be crucial for the development of calcification, is immediately impacted by alterations in blood flow [51,52]. An increased left ventricular load that is congruent with clinical symptoms is caused by the stiffening of the leaflets, which restricts the valve opening and reduces the blood transport efficiency [53]. Due to its low values, which are comparable to those of the blood vessel atherosclerosis formation predictor, the WSS in aortic valve leaflets may one day be utilized as a predictor of the onset and progression of aortic valve disease.

The primary difficulty in establishing a clear relationship between wall shear stress (WSS) and aortic valve leaflet pathology lies in the inherent complexity of both the valve’s hemodynamic environment and its biological response [54]. WSS patterns on the leaflets are highly dynamic, spatially heterogeneous, and influenced by multiple factors, including the patient-specific anatomy, flow conditions, and alterations in the leaflet geometry [55]. Moreover, the biological processes underlying the valve pathology—such as cellular inflammation, extracellular matrix remodeling, and calcific nodule formation—are multifactorial and not solely governed by local hemodynamic stress [56,57]. As a result, correlating a single mechanical parameter like WSS with specific pathological changes is challenging. Confounding influences, ranging from individual patient variability and disease stage to comorbidities and genetic predispositions, further complicate the task [58]. Thus, while WSS is likely an important contributor to disease progression, isolating its role and determining a definitive causal relationship require integrated approaches that combine high-fidelity fluid–structure interaction modeling, advanced imaging techniques, and in-depth biological and histopathological analyses.

### 4.4. Clinical Feasibility

The development of image-based CFD models toward clinical viability serves as the driving force behind our proposed computational methodology. The necessary working hours, computation time, and computational costs, in addition to data accessibility and model quality, all have an impact on the potential clinical viability of our methodology. At the designated number of CPUs, the computation time per cycle leads to rather significant computational expenses. These expenses would be decreased by combining ML approaches with synthetic database applications. When these factors are taken into account, our proposed computing framework produces pre-processing, solution, and post-processing orders of magnitude that fall within the realm of practical feasibility.

## 5. Conclusions

This study demonstrates the feasibility and robustness of our approaches to modeling both the aortic valve and the left ventricle, bridging parameterized geometric variations with patient-specific reconstructions derived from MSCT data. By systematically exploring a range of geometric parameters—such as valve leaflet dimensions and left ventricular thickness, radius, and height—we have shown that our synthetic models can reproduce physiologically relevant configurations suitable for hemodynamic analyses. These parameterized models support the validation and refinement of computational methods aimed at predicting flow behavior, structural responses, and the impact of various pathological conditions or interventional procedures.

When compared to findings in the broader literature, our results align with the typical ranges reported for healthy and diseased aortic valves, as well as normal and remodeled ventricular geometries [59,60]. Similar computational frameworks have successfully integrated patient-specific data to enhance anatomical fidelity and improve the accuracy of flow simulations [61,62]. While some discrepancies in the measured dimensions highlight the importance of personalized anatomical input, the general consistency with the published data supports our methodology. Notably, many state-of-the-art modeling approaches and imaging techniques—such as refined fluid–structure interaction (FSI) models, advanced 4D Flow MRI, and machine-learning-driven geometric evaluations—further validate and complement the present work [63].

These results resonate with studies that underscore the importance of combining parameterized and patient-specific modeling strategies to capture subtle variations in tissue morphology and mechanical properties [64]. Such approaches are essential not only for understanding the native valve and ventricular function but also for designing and evaluating novel surgical interventions, prostheses, and personalized therapies. Moreover, the ability to generate large databases of synthetic geometries offers a valuable resource for systematic investigations, sensitivity analyses, and data-driven discovery.

In conclusion, the methods detailed here provide a robust foundation for integrating simplified parametric geometries and patient-specific reconstructions, advancing the computational simulation landscape in cardiovascular research. As emerging imaging modalities, computational algorithms, and biomechanical models continue to evolve, these integrated approaches will play an increasingly pivotal role in elucidating complex hemodynamic phenomena and guiding patient-specific treatment strategies.

## Figures and Tables

**Table 1:** Dimensions of left ventricular geometry models.

No.	Height, mm	Thickness, mm			Radius, mm		
		Min	Max	Avg	Min	Max	Avg
0	80	3.36	9.96	4.58	21	37.5	31.2
1	73	3.06	10.32	4.57	21	37.5	31.1
2	75	3.11	10.38	4.53	21	37.5	31.2
3	77	3.23	10.39	4.58	21	37.5	31.2
4	79	3.3	10.29	4.59	21	37.5	31.2
5	82	3.09	10.19	4.62	21	37.5	31.2
6	85	3.26	10.15	4.64	21	37.5	31.2
7	89	3.137	10.37	4.67	21	37.5	31.2
8	80	3.31	9.88	4.22	21	36.5	30.5
9	80	3.29	9.88	4.3	21	36.7	30.6
10	80	3.19	10.02	4.41	21	37	30.9
11	80	3.16	10.03	4.48	21	37.2	31.0
12	80	3.24	10.07	4.69	21	37.8	31.5
13	80	3.27	10.05	4.86	21	38.2	31.8
14	80	2.81	10.11	4.96	21	38.5	32.0
15	80	3.21	10.11	4.55	21	37.2	31.3
16	80	3.33	10.11	4.57	21	38	31.2
17	80	3.31	10.11	4.57	21	37.2	31.2
18	80	3.18	9.979	4.58	21	38	31.1
19	80	3.57	10.11	4.6	21	37.8	31.1
20	80	2.91	10.11	4.58	21	37.5	31.1
21	80	3.13	10.12	4.6	21	37	31.1

**Table 2:** Patient data: aortic valve and left ventricular dimensions.

No.	Sex	Aortic Valve		Left Ventricular			
		Radius, mm	Height, mm	Height, mm	Radius, mm	Thickness, mm	Volume, mL
1	M	11.2	12.5	84	29	10	132
2	M	12.0	13.8	85	31	11	140
3	F	10.8	10.7	78	28	9	117
4	M	13.4	12.1	82	30	10	126
5	F	11.0	11.5	75	26	8	108
6	M	12.2	10.3	80	28	11	115

**Table 3:** Comparison of key performances.

	Case No. 1: Normal State			Case No. 2: Pathology			Case No. 3: Ozaki Procedure		
	2D	3D	Other Research	2D	3D	Other Research	2D	3D	Other Research
**Peak velocity (m/s)**	1.7	1.5	1.1–1.7 *	2.75	2.8	2.5–2.9 *	1.53	1.6	–
**Mean TPG (mmHg)**	6.94	6.2	6.47 *	17.84	16.7	15 ± 4 *	6.18	5.9	6.36 *
**Maximum 1st principal stress (kPa)**	85.5	90.4	–	77.8	80.3	–	83.7	87.5	–
**Maximum 1st principal strain (%)**	21.5	31.4	17 **	17.5	20.1	20 **	52.9	40.6	37 **
**Average WSS (Pa)**	20.4	15.8	15–21.3 *	34.6	30.2	25 **	23.8	17.1	–

*—in vivo and in vitro experimental data, **—numerical experiment data.

## Data Availability

Data are contained within the article.
